# The Spatial and Temporal Repeatability of Genomic Responses to Natural Selection as Demonstrated in Stickleback Populations Experiencing Highly Dynamic Environments

**DOI:** 10.1111/mec.70528

**Published:** 2026-09-04

**Authors:** Mary Kathleen Hickox, Ben A. Wasserman, Alan Garcia‐Elfring, Eric P. Palkovacs, Andrew P. Hendry, Rowan D. H. Barrett

**Affiliations:** ^1^ Department of Biology, Redpath Museum McGill University Montreal Quebec Canada; ^2^ Department of Ecology and Evolutionary Biology University of California Santa Cruz California USA; ^3^ Delaware Division of Fish and Wildlife Dover Delaware USA

**Keywords:** local adaptation, natural selection, parallel evolution, repeated evolution, tape of life, threespine stickleback

## Abstract

The evolution of genotypic parallelism under shared environmental conditions provides strong evidence for the role of natural selection. However, analyses typically examine genomic signatures of selection long after the putative selection event and only assess the repeatability of responses across spatial population replicates. This impedes our ability to attribute a particular response to a given selection pressure and to distinguish non‐parallel responses caused by stochastic processes from those caused by local selection. As such, the consistency of natural selection over space and time is unknown, and the role of persistent local selection pressures is unclear. Here, we leveraged the natural bar‐built estuary system of Santa Cruz, California, to examine the repeatability of seasonal genomic change in threespine stickleback ( *Gasterosteus aculeatus* ) over space and time. By comparing allele‐frequency shifts that are shared across locations (spatial repeatability) with those that are shared across years within locations (temporal repeatability), we identified both spatially shared and local components of putative selection. We found that repeated seasonal outlier responses occurred more often than expected under a neutral null model. Although repeatability declined as the number of estuaries sharing an outlier increased, enrichment above neutral expectations increased with broader spatial sharing, particularly for outliers repeated across both years. While the precise outlier SNPs varied across years, estuary‐specific patterns of responses were broadly consistent, suggesting an important role for local conditions. Together, our findings show that temporal sampling can reveal components of putative selection that would be missed from spatial comparisons alone. More broadly, they highlight the importance of examining repeatability over both space and time to understand the parallel and non‐parallel components of adaptive genomic change.

## 1 Introduction

Evolution by natural selection occurs when selective pressures act on heritable variation, resulting in differential survival and reproductive success within a population. As natural selection has long been the central pillar of evolutionary theory, many studies have examined phenotypic and/or genetic differentiation to detect signatures of selection (Bergland et al. 2014; Dudaniec et al. 2018; Henkel et al. 2019), where repeated patterns are often interpreted as support for a deterministic (i.e., predictable) effect of natural selection. A common approach to inferring such selection in nature is to assess the genetic or phenotypic differentiation of populations or species that occupy environments differing in one or more selective forces, such as predator presence/absence (Langerhans 2018), acidic versus basic habitats (Haenel et al. 2019), or different altitudes (Bohutínská et al. 2021). These genetic or phenotypic differences can be assessed in multiple pairs of populations that have evolved independently (Berner et al. 2008; Elmer et al. 2014; Reid et al. 2016). The degree to which environment‐related population differences are shared across these evolutionary replicates then suggests the extent to which selection is spatially repeated and thus how similar environments drive similar (i.e., parallel, convergent or predictable) evolutionary responses (Barghi et al. 2020; Bolnick et al. 2018). Although such spatially repeated signatures can reflect shared selection, they leave open the question of how many responses are not shared among environments but instead arise from local selection, stochastic processes, or population‐specific genetic backgrounds. As a result, we still have a limited understanding of how much of the total genomic response is consistently shared across similar environments versus how much is specific to local conditions.

Many analyses focus on inferring past selection by assessing differences in contemporary populations in relatively stable environments (e.g., Zhang et al. 2022). Such studies are examining the outcome of adaptive divergence, rather than selection itself. There are two limitations to this approach. First, ‘selection erases its traces’ (Haller and Hendry 2014) such that well‐adapted populations are no longer experiencing the selection that caused their divergence in the first place. Second, studies of divergence in contemporary populations use spatial replicates (as described above) to infer selection that is shared across locations (e.g., Garcia‐Elfring et al. 2021; Takahashi 2026; Terekhanova et al. 2014), and they thus lack the means to infer deterministic local selection. We suggest that these limitations can be addressed by measuring the temporal (e.g., across years) repeatability of allele‐frequency change in highly dynamic environments, where recurrent environmental shifts might generate repeated seasonal responses. By comparing allele‐frequency shifts that are shared across locations (spatial repeatability) with those that are shared across time within locations (temporal repeatability), one could gain insight into both the spatially shared and the local components of putative selection. The need for such studies has been consistently expressed in the field (Barghi et al. 2020; Jackson et al. 2026; Pfenninger and Foucault 2022). Here, we examine the spatial and temporal repeatability of seasonal genomic responses in the threespine stickleback ( *Gasterosteus aculeatus* ) populations occupying bar‐built estuaries in Santa Cruz, California.

Bar‐built estuaries along the coast of Santa Cruz represent a powerful system to study selection within and across highly variable environments. The estuaries close during the spring or summer when wave action and reduced precipitation result in the formation of sandbars that isolate the estuaries from the Pacific Ocean (Behrens et al. 2009, 2013; Orescanin and Scooler 2018). During this time, stickleback are trapped in the estuaries and thus experience dramatic environmental shifts towards more pond‐like conditions. Such shifts include salinity stratification in the water column (Behrens et al. 2016; Bond et al. 2022), an increase in aqueous pH levels and a decrease in hydrogen sulfide (Richards et al. 2018), a decrease in water movement (Des Roches et al. 2020), and shifts in predator abundance (Frechette et al. 2016). Then, in the late fall or winter, higher precipitation results in increased streamflow (Behrens et al. 2009, 2013; Orescanin and Scooler 2018) that can breach the sandbars and reopen the estuary to the ocean. These breaching events generally occur very rapidly over a period of minutes to hours. During breaching events, most stickleback are washed out to sea and thus experience both marine and freshwater‐like conditions within a single generation, likely reducing population structure while increasing within‐population variation (Paccard et al. 2018). After several days to months, reduced rainfall allows wave action to reform the sandbar, trapping many new breeding stickleback in the estuary (Wasserman et al. 2022).

As a result of these processes, the stickleback occupying the bar‐built estuaries experience extreme yet predictable shifts in environmental conditions that are replicated over space (i.e., multiple estuaries) and time (i.e., annual breaching), with substantial allelic reshuffling expected across the system. These environmental shifts involve variables, including salinity, predation, and diet that are known to impose strong selection on stickleback (Gross 1978; Heckwolf et al. 2018; Leinonen et al. 2011), and therefore provide plausible recurrent selective pressures in this system. Because many ecologically relevant traits in threespine stickleback have substantial genetic components (Des Roches et al. 2020; Glazer et al. 2014; Shapiro et al. 2004), at least some seasonal responses might be detectable as genomic allele‐frequency change. Further, the open phase likely increases gene flow among estuaries, and between estuaries and marine habitats (Paccard et al. 2018). This seasonal mixing might reduce persistent differentiation and thereby increase our ability to detect within‐season allele‐frequency change, although it might not fully homogenize the populations. We therefore interpret this system as providing an opportunity to study seasonal genomic change consistent with selection in real‐time. Supporting this inference, our previous work has shown genome‐wide seasonal allele‐frequency change within a generation across multiple estuaries based on a single year of analysis (Garcia‐Elfring et al. 2021). In the present work, we extend these previous methods to examine not just the spatial repeatability of selection but also the temporal repeatability, thus providing insight into selection that might be deterministic within specific locations. We predict that much selection is shared over space and/or time, and we expect that the selective landscape is composed of both local and shared selective responses. Given the environmental heterogeneity and temporal variability of the selective landscape, high gene flow, high genetic variance, and short duration of the selective events (i.e., within a generation), we expect the responses to be highly polygenic, rather than encompassing a few tightly linked regions of large effect (Ehrlich et al. 2021; Garcia‐Elfring et al. 2021; Pfenninger and Foucault 2022; Yeaman 2015).

We sampled threespine stickleback at the beginning and end of the period in which the estuaries are closed (our period for estimating selection) in six estuaries in both 2016 and 2017. We then used pooled whole‐genome sequencing and calculated spring‐to‐fall F_ST_ to estimate selection across the genome in each of these 12 samples: a single selective episode in each of the two years for all six estuaries. Because we sampled only adults, our analyses are intended to capture allele‐frequency changes within a generation rather than differences generated by reproduction during the sampling interval. We analysed the resulting data to assess the repeatability of selection over space and time corresponding to various repeatability ‘types’ (Table S1). Specifically, we ask:

1. How repeatable is natural selection over space and time?
2. How do the genomic targets of selection and their associated functions vary by repeatability type?

## 2 Methods

### 2.1 Sample Collection

**FIGURE 1:**
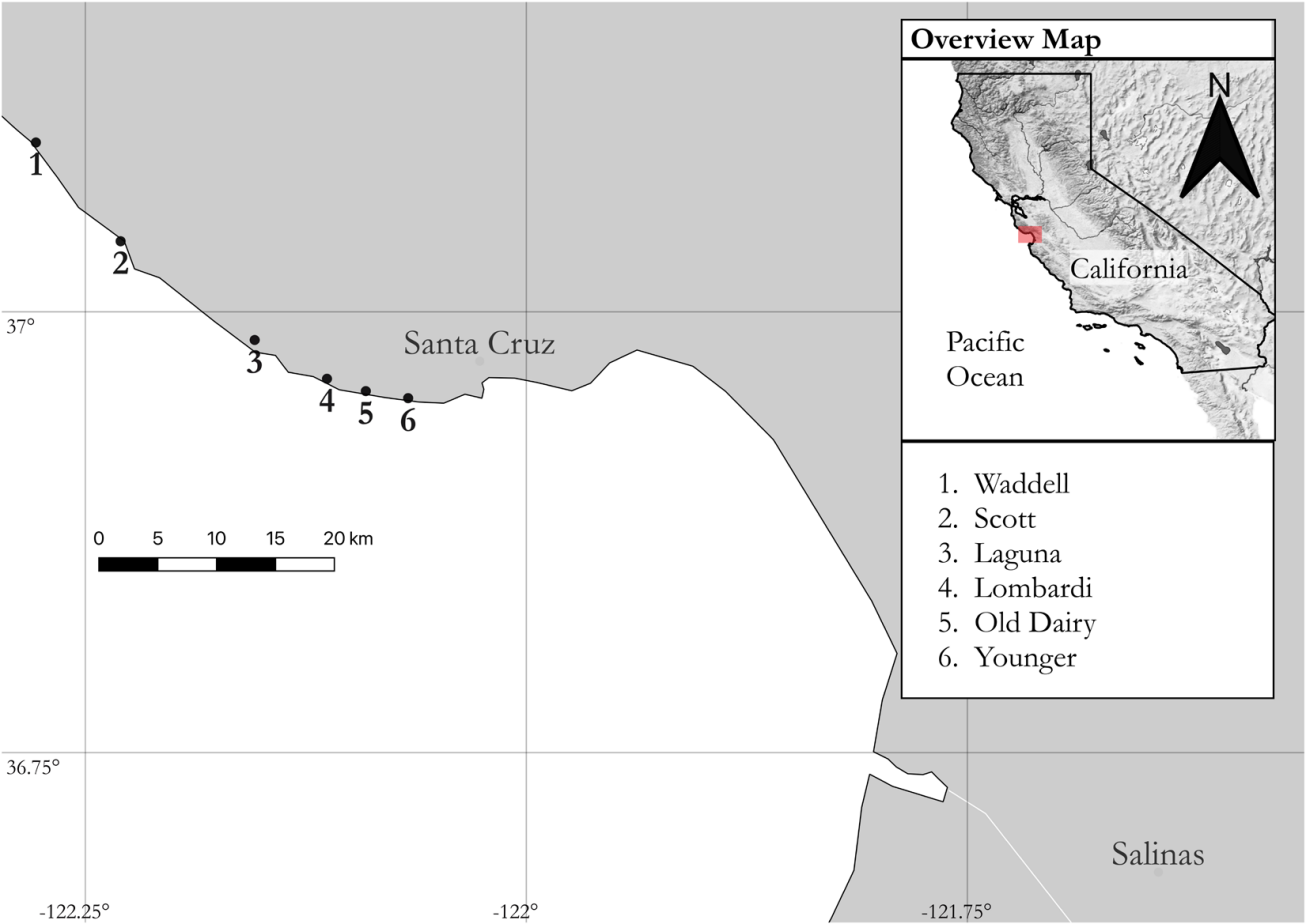
Map of the estuaries sampled. Each estuary was sampled in both the spring and fall of 2016 and 2017. The red square in the overview map depicts the sampling region.

Between 2016 and 2017, we performed sampling four times (spring and fall in each of the two years) in six bar‐built estuaries: Old Dairy, Younger, Scott, Laguna, Lombardi, and Waddell (Figure 1). Spring and fall sampling occurred in May–July and November–December, respectively. The specific timing of sampling varied among estuaries based on the time of breaching (which is estuary‐specific), so that the spring samples were collected just after sandbar formation, and the fall samples were collected just before sandbar breaching. The sampling period thus represents the time during which the estuaries were isolated from the ocean, and the stickleback were restricted to their respective estuaries. During this period, the estuaries transition from marine‐like to pond‐like, thus encompassing our putative selective regime. Because our analyses focus on spring‐to‐fall allele‐frequency change within each estuary, the spring sample serves as the relevant baseline for testing repeatability during the seasonal period of isolation. Oceanic samples would be informative for assessing ancestry, connectivity and absolute divergence from marine populations, but they are not required to test our primary question. We only collected fish above 30 mm in standard length, so that any putative responses to selection represent differential survival, rather than differential reproductive success. We used minnow traps and tricaine methanesulphonate (MS‐222) to catch and euthanize fish.

### 2.2 Environmental Measurements

At each sample site and collection period, we used a YSI Pro2030 meter to measure dissolved oxygen (mg/L), salinity (ppt), and temperature (°C) at the water surface. We averaged the data per population, before running a principal component analysis (PCA) for each year. We generated the PCA on the centered and scaled data using prcomp, R v.4.4.0, before visualizing the biplots using ggplot2 v.3.5.1, and factoextra v.1.0.7. We also collected precipitation data (cm) for each sampling site from the closest National Weather Service station (Table S2).

### 2.3 Molecular Work and Sequencing

We extracted DNA from the pectoral and gill tissues using a standard phenol‐chloroform protocol with a proteinase‐K digestion at 55°C and an ethanol wash. We resuspended the DNA in Tris‐EDTA and quantified it using the Quant‐iT dsDNA High‐Sensitivity PicoGreen assay kit (Thermo Fisher Scientific, Waltham, MA, USA). Samples with concentrations below 25 ng/μL were reconcentrated with a Savant SpeedVac (Thermo Fisher Scientific, Waltham, MA, USA), and requantified. We normalized and requantified all samples before pooling equal quantities of DNA according to the collection year, season, and estuary of origin. Each pool contained between 21 and 40 individuals (Table S3). The McGill Genome Center, Montreal, Canada, then prepared our 24 libraries using PCR‐free, Lucigen NxSeq AmpFREE kits (Lucigen, Middleton, WI, USA) for pooled whole‐genome sequencing at targeted 50× coverage. The 2016 samples were sequenced across five lanes of Illumina HiSeq2500 with paired‐end, 125 bp reads, while the 2017 samples were sequenced across one lane of NovaSeq6000, with paired‐end, 150 bp reads.

### 2.4 Bioinformatics

We assessed the quality of the raw FASTQ files using FastQC v.0.12.0 and MultiQC v.1.16 (Andrews 2010; Ewels et al. 2016). Using FastP v.0.23.4 (Chen et al. 2018), we then trimmed the reads from the tail to the front, removing adapter sequences, polyG tails, reads with phred scores below 20, and reads with lengths below 50 base pairs. Only paired‐end reads meeting these criteria were retained. We then reran FastQC and MultiQC (Andrews 2010; Ewels et al. 2016) to confirm adequate filtering. The reads were then aligned to the University of Georgia (UGA) *G. aculeatus* reference genome, version 5 (Nath et al. 2021) using BWA‐MEM v.0.7.17 (Li 2013). We converted, sorted and merged the resulting bam files using SAMtools v.1.17 (Li et al. 2009), requiring a mapping quality of 20 or greater. We removed PCR duplicates and converted the data to an mpileup file using Picard Tools v.2.26.3 (Broad Institute 2019) and SAMtools v.1.17 (Li et al. 2009), respectively. We filtered for a minimum depth of coverage of five reads, identified regions 5 bp above and below indels, created a sync file filtering for base quality above 20 and masked the indel regions using Popoolation2 v.1.201 (Kofler et al. 2011). We retained all autosomes, removing scaffolds, chromosome 19 and mitochondrial sequences and used the *popcov* function of popoolation2helper (Wang 2020) to calculate the depth of coverage at each genomic site across both years separately.

### 2.5 Calculating Seasonal Genomic Differentiation

As in prior work in this system (Garcia‐Elfring et al. 2021), we used F_ST_ to measure changes in allele frequency within a lineage across time points, rather than differentiation between two lineages (Burri 2017). For each year, we used the poolfstat v3.0.0 functions of *popsync2pooldata* and *compute.pairwiseFST* (method = Anova) to generate poolitems and the pairwise F_ST_ values (Gautier et al. 2022; Hivert et al. 2018). Using the same program, we also ran a sliding window analysis to assess whether the SNP‐level patterns were supported at broader genomic scales (*sliding.windows.fstat*, sliding.window.size = 50,000, window.overlap.fact = 0.2, bp.start.first.snp = FALSE). For both years, we conducted a genomic site‐level analysis using the filtering parameters described below. Single‐nucleotide polymorphisms (SNPs) were retained only when the minor allele count was at least two and each pool had a coverage of at least five reads (min.rc = 2, min.cov.per.pool = 5) (Garcia‐Elfring et al. 2021). To account for year‐specific differences in read‐depth distributions, we set the maximum coverage limit to 100 and 300 for the 2016 and 2017 datasets, respectively. We then calculated the pairwise F_ST_ values between spring and fall for each estuary‐year contrast, so that high F_ST_ values indicate large seasonal allele‐frequency shifts. We interpret these high F_ST_ outliers as candidate signals of putative selection, rather than as definitive evidence that individual SNPs are causal targets of selection. Using the tidyverse v.2.0.0 and datatable v.1.16.4 packages in R v.4.4.0, we restricted the datasets to only include genomic sites present in both years. As populations fixed on the major allele and those with negative F_ST_ values are biologically interpreted as having no genetic differentiation (e.g., Haro‐Bilbao et al. 2021), we transformed all ‘NA’ and negative values to zero. We defined outliers as SNPs that fell in the top 5% of the F_ST_ distribution. SNPs that were not outliers in any population were considered to be neutral.

Using the same parameters, we calculated allele frequencies with the *snp‐frequency‐diff.pl* function of Popoolation2 v.1.201. We then pruned the data to generate 2016 and 2017 datasets, containing the 5% F_ST_ outliers of each year. We converted all allele counts to frequencies using the *calfreq* function of popoolation2helper (Wang 2020). We used the *prcomp* function of R v.4.4.0 to perform a PCA on the centered and scaled data, running each year in separate ordination space. To compare the patterns of genomic response to selection across years, we generated PCAs of the top 5% F_ST_ outliers for 2016 and 2017. To minimize any potential effects of underlying population structure, we ran the PCAs on the 5% F_ST_ outliers rather than on all SNPs. As the outliers are based on within‐estuary seasonal allele‐frequency change over our putative period of selection, they should be less readily explained by static founding differences alone. We then visualized PC1 and PC2 using ggplot2 v.3.5.1 and factoextra v.1.0.7, with each panel representing a separate year. We further quantified relationships among the neutral SNPs, outlier SNPs and environmental variables using a vector analysis following Stuart et al. (2017) (see Methods S1).

### 2.6 Assessing the Repeatability of Seasonal Genomic Responses

Using the 5% F_ST_ outliers, we defined different types of repeatability over the spatial (i.e., estuaries) and temporal (i.e., 2016 and 2017) scales (Table S1). A spatially repeated SNP is an outlier in two or more estuaries in the same year, but not an outlier in any estuary in the other year. A temporally repeated SNP is an outlier in both 2016 and 2017 in the same estuary, and only for that estuary. ‘Spatio‐temporally’ repeated SNPs are outliers in both years in the same estuary for multiple estuaries. We ran custom awk and R scripts to generate repeatability datasets, before using ggplot2 v.3.5.1 (Wickham et al. 2016) and ComplexUpset (v.1.3.3, exclusive interaction, min_size = 1, min_degree = 2) (Krassowski 2020; Lex et al. 2014) to compare the frequencies and characteristics of each repeatability type. Because we used ‘exclusive interaction’, the outliers were binned by type so that they belong to only a single set of overlapping estuaries and/or time points (i.e., an outlier that is spatially shared among four estuaries is not considered to also be shared among three and/or two estuaries). We additionally ran a custom spatial clustering pipeline in R to determine whether the outlier SNPs were clustered or randomly distributed across the genome (Methods S2).

The repeatability types (i.e., spatial, temporal, spatio‐temporal) are not equally likely to occur through random overlap because they can arise through different numbers of estuary and year combinations. For example, spatial repeatability can involve any subset of two to six estuaries, giving ∑k=266k=57 possible estuary combinations in a year, or 114 combinations across two years. By contrast, temporal repeatability has six possible estuary‐specific combinations. To account for these differences, we calculated the expected occurrence of each type based on the total number of SNPs (n=6,261,838) and the expected probability of each type (Methods S3). We assumed that all genomic sites were independent and that each had a 5% chance of being an outlier. Each repeatability type had a different probability of occurring and was calculated as:

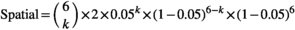

$$\text{Temporal}=6\times 0.05^{2}\times {\left(1-0.05^{2}\right)}^{5}$$

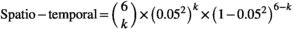

$$\begin{array}{c} \begin{array}{c} \begin{array}{c} \begin{array}{c} \text{where}\;k=2:6,\text{and represents the number of estuaries that share}\;\\ \text{an outlier}. \end{array} \end{array} \end{array} \end{array}$$

We then generated the observed‐to‐expected ratios as a standardized measure of enrichment, allowing us to compare repeatability types that differ in the number of possible combinations. To determine if each repeatability type occurred significantly more than expected by chance, we ran permutations (*n* = 1000) by randomly selecting 5% of all SNPs per population as outliers. We generated *p*‐values by summing the number of times the permutation data had an equal or greater number of occurrences than the observed data, dividing by the number of permutations, with a correction of plus one in both the numerator and denominator (Phipson and Smyth 2010). To ensure the robustness of our 5% analyses, we regenerated the probability and permutation calculations using both 1% and 10% cutoff values.

### 2.7 Candidate Genes and Functional Enrichment

We identified candidate genes in nine focal datasets that represent the major spatial, temporal and spatio‐temporal repeatability classes. To assess temporally unique but spatially widespread outlier responses, we generated two ‘highly spatial’ datasets containing SNPs that were outliers in four or more estuaries in either 2016 or 2017, but not in the other year. We also generated six estuary‐specific datasets, each containing SNPs that were outliers in both years within a single estuary. Finally, to assess spatially broad and temporally consistent responses, we generated a ‘highly spatio‐temporal’ dataset containing SNPs that were outliers in both years in three estuaries. We mapped outlier SNPs to protein‐coding genes using the UGA *G. aculeatus* annotation assembly, version 5 (Nath et al. 2021), and used all protein‐coding genes in this annotation as the reference gene universe for the enrichment analysis. Because repeatability was defined at the SNP level, and because multiple SNPs can map to the same gene, the same gene could be a candidate in multiple repeatability types. To better understand how repeatability varies at the level of loci, we calculated the average number of genes unique to each dataset. Using the biomaRt v.2.62.1 and topGO v.2.58 packages, we performed enrichment analyses of biological processes (algorithm = ‘classic’, statistic = ‘fisher’, nodeSize = 10).

## 3 Results

### 3.1 Sequencing and Assembly

The raw 2016 and 2017 datasets had 1,147,918,746 and 2,686,979,681 paired reads, respectively. After trimming, cleaning and deduplicating the bam files, we retained 142,650,392 and 332,918,604 reads in the 2016 and 2017 datasets, respectively. The mean read depth for the sync file was 34.38 in 2016 and 86.89 in 2017, and we called 6,261,838 SNPs present in both datasets.

### 3.2 Repeatability of Seasonal Genomic Responses

**FIGURE 2:**
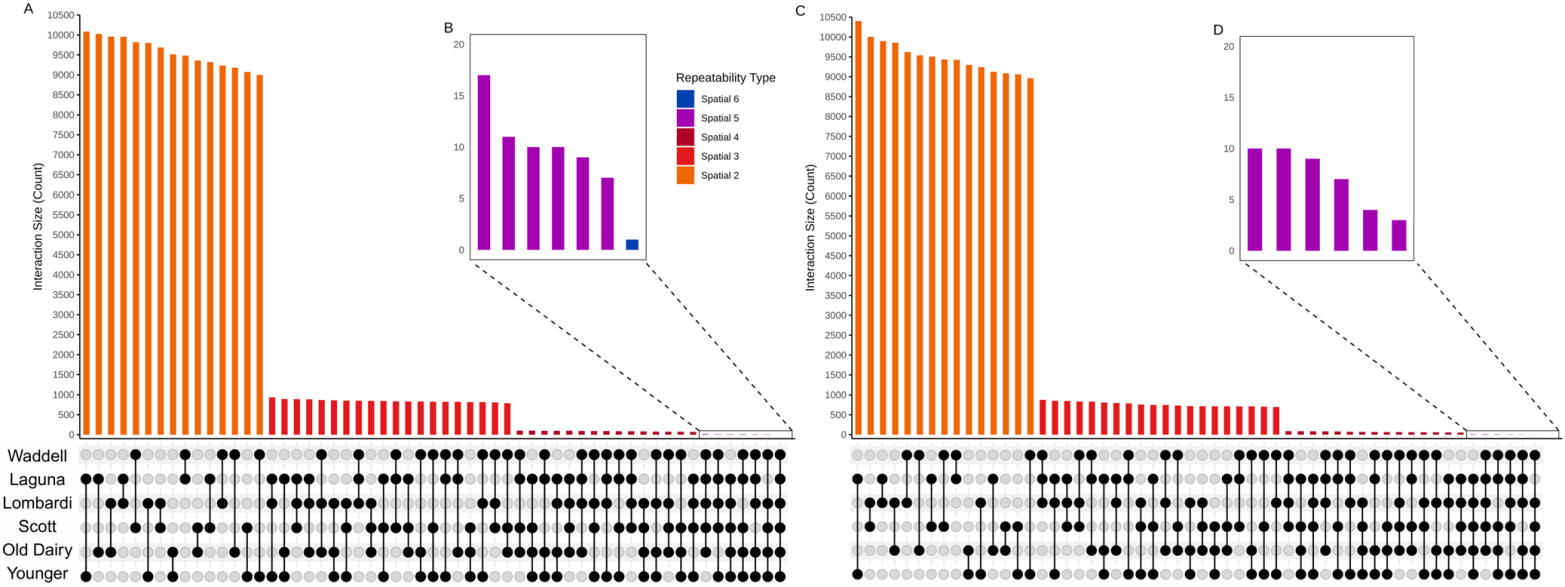
UpSet plots showing the number of spatially‐repeated outliers across all estuaries in (A) 2016 and (C) 2017, with the magnified results over five and six estuaries shown in (B, D), for each year. Interactions are coloured by the degree of spatial repeatability, defined as the number of estuaries sharing an outlier within a year. The intersections are mutually exclusive, so that each outlier belongs to only one set of overlapping estuaries.

**FIGURE 3:**
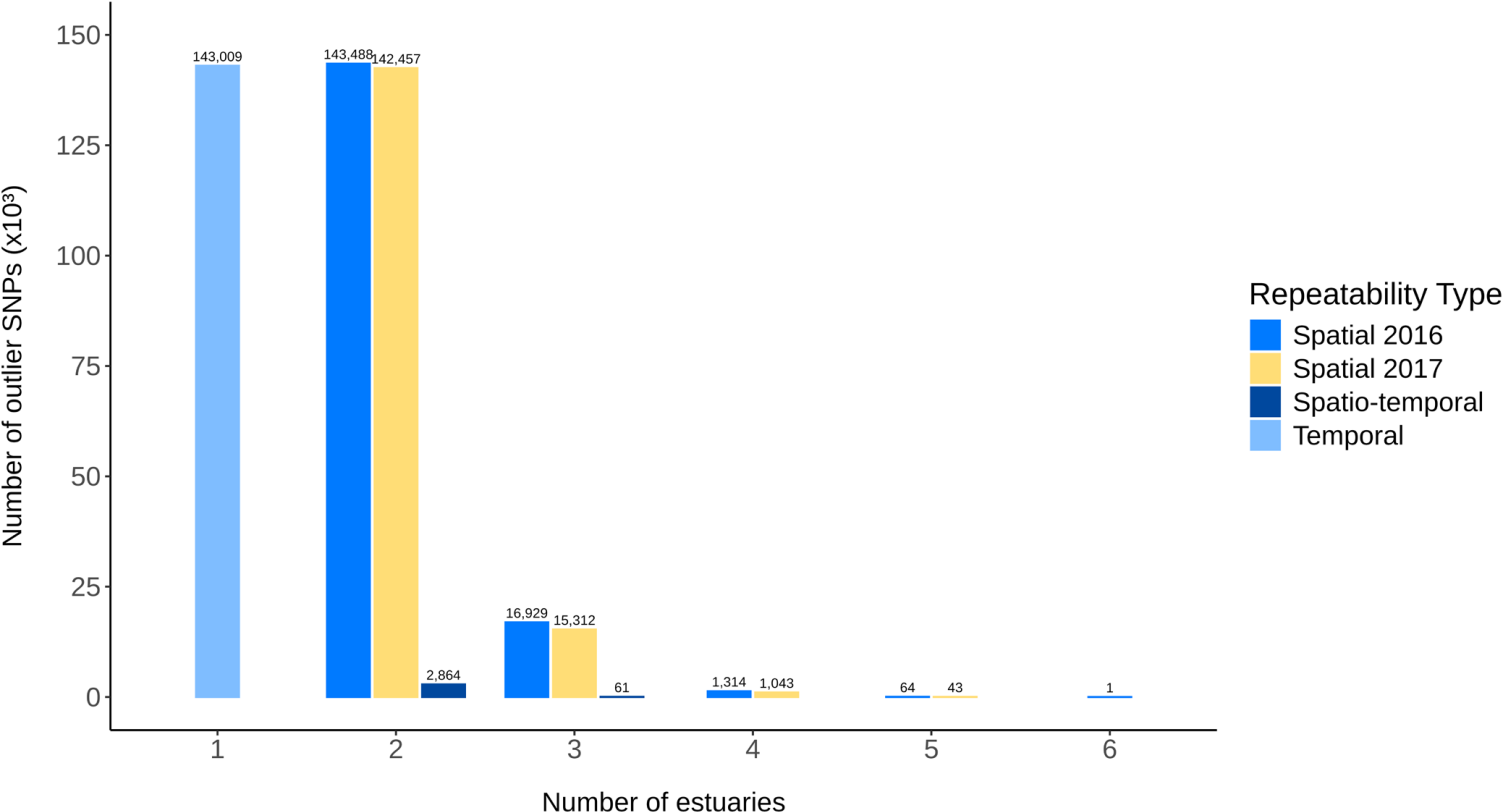
The number of outlier SNPs per repeatability type. The *x*‐axis outlines the number of estuaries for which each SNP is an outlier. The number of SNPs for each repeatability type is listed above the associated bar.

To examine how repeatability varies over space and time, we calculated the occurrences of each repeatability type (i.e., spatial, temporal, spatio‐temporal). We detected 161,796 spatially‐repeated outliers in 2016 and 158,855 in 2017. As expected, there were fewer outliers shared between a greater number of estuaries, with only a single outlier SNP shared between all six estuaries (Figure 2B). In 2016, Laguna and Lombardi appeared to share many outliers with other estuaries, while Waddell and Scott appeared to share many outliers with each other (Figure 2A). Fewer discernible patterns of sharing among estuaries were detectable in 2017 (Figure 2C). Approximately the same number of temporally‐repeated outliers were detected within a single estuary as were spatially shared between pairs of estuaries within a year (~143,000; Figure 3). Spatio‐temporally repeated outliers were shared across either two or three estuaries, but none were shared across four, five, or six estuaries (Figure 3). Within both the spatial and spatio‐temporal categories, observed counts declined as the number of estuaries sharing an outlier increased (Table 1). All categories with at least one observed outlier occurred more frequently than expected by chance, except for spatial 6 (Table 1). In contrast to the raw counts, observed‐to‐expected enrichment generally increased with broader spatial sharing (Table 1). These qualitative patterns were generally robust to 1% and 10% cutoff values, although spatial 2 was not significantly enriched under the 10% cutoff (Table S4).

**TABLE 1:** The repeatability types used in the analysis. The numeric value indicates the number of estuaries involved; for example, ‘Spatial 2’ refers to spatial repeatability across two estuaries. ‘Observed’ indicates the number of occurrences. ‘Ratio’ is a standardized measure of enrichment, calculated as the ratio of observed occurrences to those expected by chance, based on probability calculations. The *p*‐value is based on 1000 permutations.

Repeatability type	Observed	Ratio	Permutation *p*‐value
Spatial 2	285945	1.02	< 0.001
Spatial 3	32241	1.63	< 0.001
Spatial 4	2357	3.03	< 0.001
Spatial 5	107	6.53	< 0.001
Spatial 6	1	6.95	0.154
Spatio‐temporal 2	2864	4.93	< 0.001
Spatio‐temporal 3	61	31.41	< 0.001
Spatio‐temporal 4	0	0	1.000
Spatio‐temporal 5	0	0	1.000
Spatio‐temporal 6	0	0	1.000
Temporal	143009	1.54	< 0.001

### 3.3 Consistency of Patterns of Seasonal Responses

**FIGURE 4:**
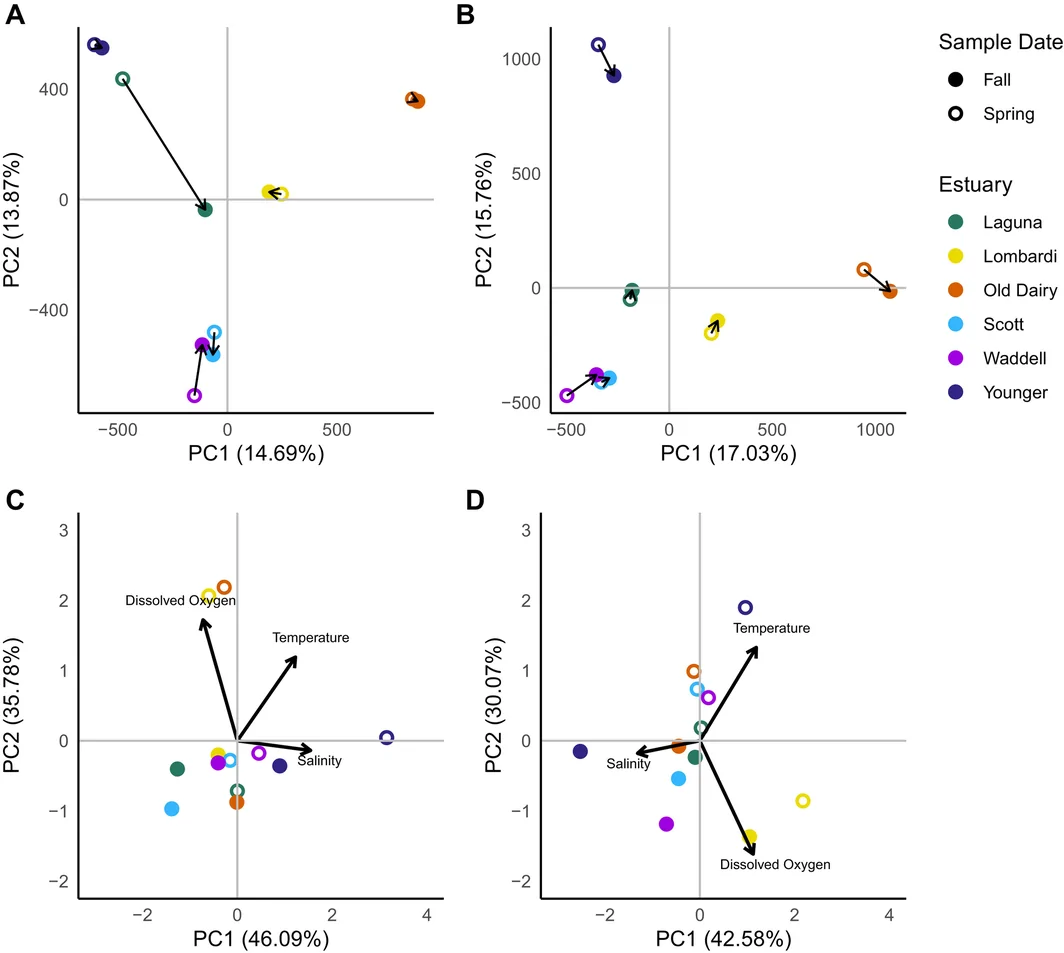
PCA of (A) SNP outliers detected in 2016 (B) SNP outliers detected in 2017 (C) Averaged environmental data from 2016 (D) Averaged environmental data from 2017. Each colour represents a distinct estuary, and the fill represents the collection date. In panels A and B, arrows show the seasonal transition from spring to fall within each estuary. The PCAs were fit separately for each year, so that the axes are not directly comparable between years. The percentage variance explained in PC1 and PC2 is shown on the *x* and y axes, respectively.

To explore the similarity of genome‐wide patterns of responses to selection over time, we generated PCAs of the outliers in both years. Together, PC1 and PC2 explained 28.56% and 32.79% of the variance for 2016 and 2017, respectively (Figure 4A,B). The patterns of differentiation between estuaries are similar in 2016 (Figure 4A) and 2017 (Figure 4B). For example, in both years, Waddell and Scott are positioned closely to each other (Figure 4). Likewise, Laguna and Lombardi are positioned most closely to each other, and approximately equally distant from all other estuaries, while Younger is positioned most distantly from the other estuaries (Figure 4). In addition, in both years, the spring and fall samples of each estuary tend to be closer to each other than to samples from the same season but different estuaries (Figure 4).

To characterize the measured environmental variation over space and time, we ran a PCA on the environmental data from each estuary in each year (Figure 4C,D). PC1 and PC2 explained a total of 81.87% and 72.65% of the variance for 2016 and 2017, respectively (Figure 4C, Figure 4D). The largest loadings on PC1 were salinity (0.74, −0.63) and temperature (0.58, 0.57) in both years (Figure 4). Those of PC2 were dissolved oxygen (0.82, −0.77) and temperature (0.57, 0.63) (Figure 4). We did not detect any significant relationships in the environmental‐genetic vector analysis (Table S5).

### 3.4 The Genomic Distribution of Seasonal Genomic Responses

**FIGURE 5:**
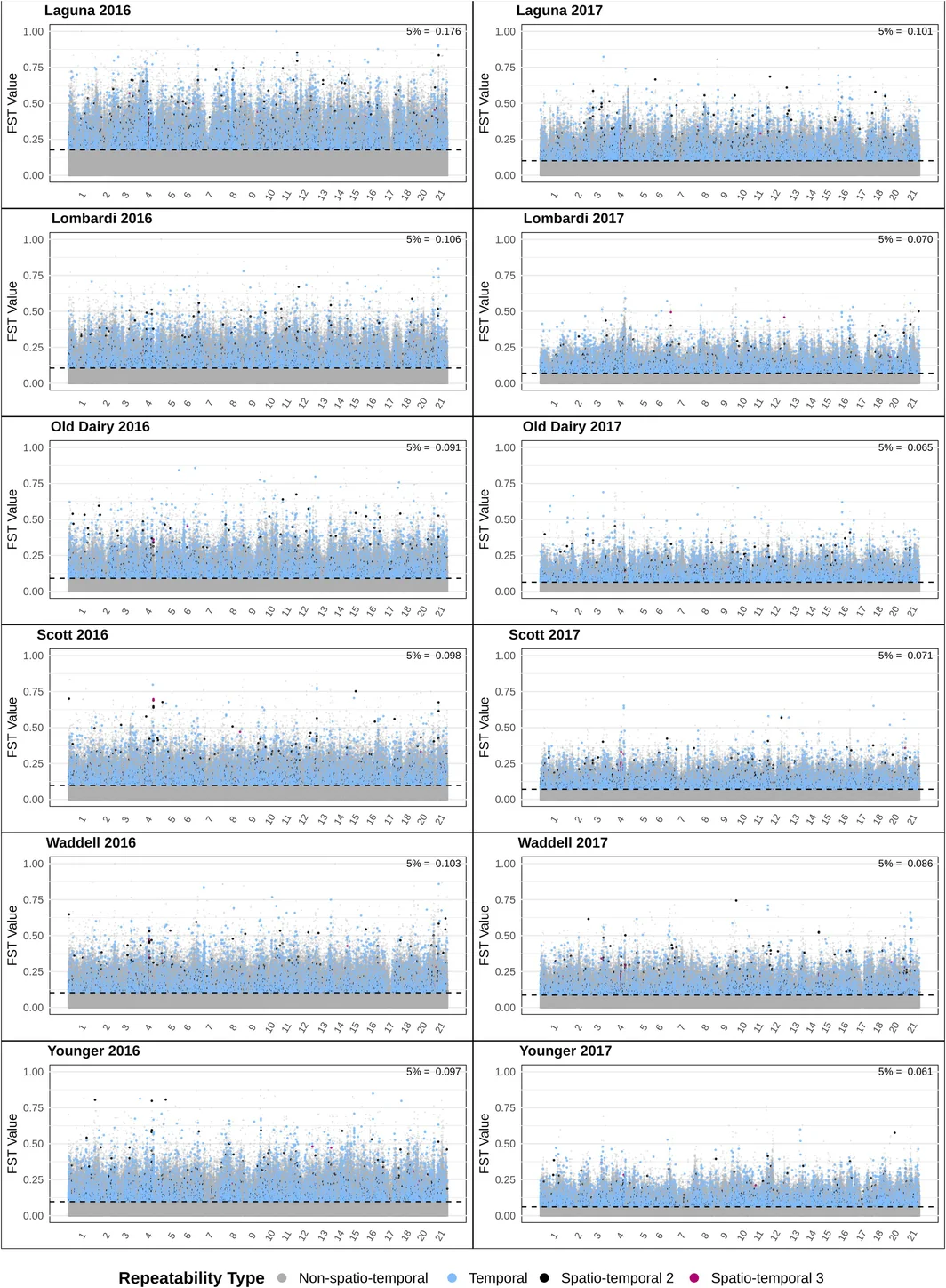
Manhattan plots showing F_ST_ values across the genome in each estuary/year. The 5% cutoff value is represented by the dashed line, so that genomic sites above the line are outliers. The genomic sites are coloured according to repeatability type for the temporal and spatio‐temporal outliers. ‘Temporal’ refers to genomic sites that are outliers in both years for a single estuary. ‘Spatio‐temporal 2’ and ‘Spatio‐temporal 3’ represent genomic sites that are outliers in both years for two and three estuaries, respectively.

To examine how outlier responses were distributed across the genome for spatial (Figure S1), temporal (Figure 5), and spatio‐temporal (Figure 5) repeatability types, we generated Manhattan plots of the F_ST_ values (*n* = 6,261,838) for all samples. The outliers were broadly distributed across the genome in both the SNP (Figure S1, Figure 5) and the sliding‐windowed analyses (Figure S2). Across populations and chromosomes, most outlier sets did not show detectable clustering, although a small number showed weak clustering in the Ripley's K analyses (Table S6). The Manhattan plots also showed a visually prominent concentration of spatio‐temporally repeated outliers on chromosome 4 in Laguna, Old Dairy, Scott, and Waddell (Figure 5), although formal support for clustering in this region was mixed. Across all estuaries, the F_ST_ values were lower in 2017 than in 2016 (Figure 5), with mean 5% F_ST_ cutoff values of 0.0756 and 0.112, respectively. The F_ST_ cutoff value was highest in Laguna for both years (Figure 5). The seasonal difference in absolute allele frequency differences followed a similar pattern with means of 0.273 (sd = 0.0905) and 0.209 (sd = 0.0729) for the 2016 and 2017 outliers, respectively.

### 3.5 Candidate Genes and Functional Enrichment

**FIGURE 6:**
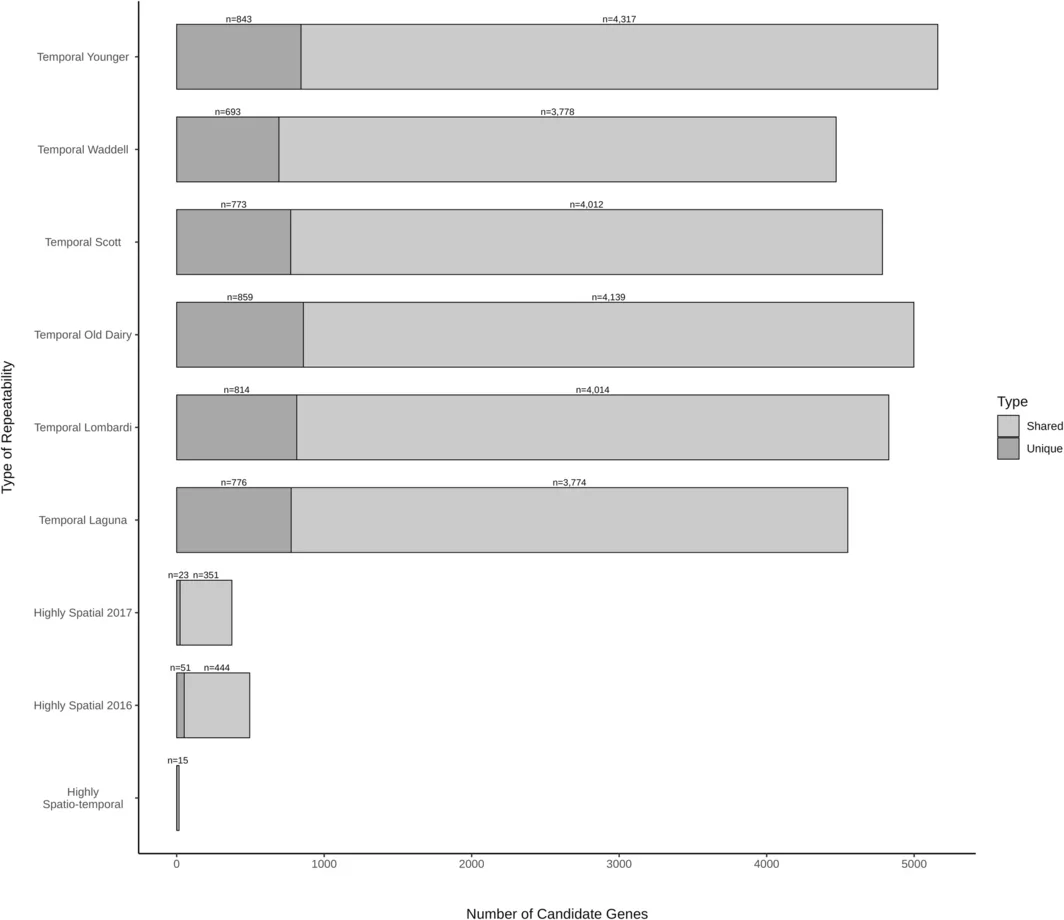
Bar plot demonstrating the number of candidate genes for each repeatability type. Unique genes are those that are candidates exclusively in the associated repeatability type, whereas those that are candidates in multiple repeatability types are ‘shared’. ‘Highly Spatial’ refers to outliers detected in four to six estuaries in either 2016 or 2017, whereas ‘Temporal’ refers to outliers detected in both 2016 and 2017 in each estuary, exclusively (i.e., local). ‘Highly spatio‐temporal’ refers to outliers in 2016 and 2017 in three estuaries. One unique gene is highly spatio‐temporally repeated (not listed).

Across all nine datasets (see Methods), we detected 60,950 genic matches representing 12,624 different IDs (Tables S7–S15), with duplicate matches within and across the datasets. No gene was detected in all datasets, but many genes were shared across multiple datasets (Tables S7–S15), with considerable sharing between the temporal and spatial datasets. On average, 13.53% (sd = 4.66) of candidate genes were unique to each repeatability type, with the remaining being candidate genes shared across multiple repeatability types (Figure 6). After identifying candidate genes (Tables S7–S15), we performed functional enrichment analyses for all repeatability types (Table S16), focusing on the top three per type (Table 2). No genes in the ‘highly spatio‐temporal’ dataset mapped to any gene ontology (GO) terms. While certain functions were unique to a particular dataset (e.g., organic acid metabolic activity in Old Dairy and appendage‐related development in Laguna), many functions were consistently detected across types. For example, GO terms related to nervous system development, cell migration/organization and anatomical/tissue development were detected across multiple repeatability types.

**TABLE 2:** The gene ontology (GO) IDs, term, number of annotated genes and *p*‐value for the top three GO IDs per repeatability type.

Repeatability type	GO ID	Term	Annotated	Significant	Expected	Classic *p*‐value
Highly Spatial 2016	GO:0050877	Nervous system process	28	5	0.81	8.5 × 10^⁻4^
Highly Spatial 2016	GO:0030855	Epithelial cell differentiation	46	5	1.33	8.2 × 10^⁻3^
Highly Spatial 2016	GO:0009888	Tissue development	279	14	8.05	1.23 × 10^⁻2^
Highly Spatial 2017	GO:0007411	Axon guidance	26	3	0.5	1.2 × 10^⁻2^
Highly Spatial 2017	GO:0097485	Neuron projection guidance	26	3	0.5	1.2 × 10^⁻2^
Highly Spatial 2017	GO:0048468	Cell development	262	10	5.04	1.2 × 10^⁻2^
Temporal Laguna	GO:0048646	Anatomical structure formation involved in morphogenesis	142	54	37.93	8.9 × 10^⁻4^
Temporal Laguna	GO:0016331	Morphogenesis of embryonic epithelium	12	8	3.21	4.13 × 10^⁻3^
Temporal Laguna	GO:0009653	Anatomical structure morphogenesis	356	112	95.09	6.46 × 10^⁻3^
Temporal Lombardi	GO:0034330	Cell junction organization	19	12	5.7	2.6 × 10^⁻3^
Temporal Lombardi	GO:0003008	System process	70	32	21.01	2.9 × 10^⁻3^
Temporal Lombardi	GO:0070727	Cellular macromolecule localization	15	10	4.5	3.4 × 10^⁻3^
Temporal Old Dairy	GO:0009653	Anatomical structure morphogenesis	356	126	109.54	1 × 10^⁻2^
Temporal Old Dairy	GO:0048048	Embryonic eye morphogenesis	12	8	3.69	1.1 × 10^⁻2^
Temporal Old Dairy	GO:0048596	Embryonic camera‐type eye morphogenesis	12	8	3.69	1.1 × 10^⁻2^
Temporal Scott	GO:0001755	Neural crest cell migration	16	11	4.41	6.4 × 10^⁻4^
Temporal Scott	GO:0090497	Mesenchymal cell migration	16	11	4.41	6.4 × 10^⁻4^
Temporal Scott	GO:0009888	Tissue development	279	92	76.9	1.03 × 10^⁻2^
Temporal Waddell	GO:0048858	Cell projection morphogenesis	58	29	15.8	1.2 × 10^⁻4^
Temporal Waddell	GO:0061564	Axon development	58	29	15.8	1.2 × 10^⁻4^
Temporal Waddell	GO:0007409	Axonogenesis	54	27	14.71	2.1 × 10^⁻4^
Temporal Younger	GO:0001755	Neural crest cell migration	16	12	5.11	4.6 × 10^⁻4^
Temporal Younger	GO:0090497	Mesenchymal cell migration	16	12	5.11	4.6 × 10^⁻4^
Temporal Younger	GO:0016477	Cell migration	65	33	20.76	8.4 × 10^⁻4^

## 4 Discussion

Threespine stickleback are a model organism for understanding parallelism and repeatability at the genomic level, and thus for inferring the predictability, or ‘determinism’, of natural selection (Kaeuffer et al. 2012; Liu et al. 2018; Magalhaes et al. 2021). However, nearly all such studies use contemporary patterns of population divergence to infer signatures of past selection that could have occurred hundreds to thousands of years (Coll‐Costa et al. 2024; Dahms et al. 2024; Hudson et al. 2021; Magalhaes et al. 2021), or decades (Lescak et al. 2015) after the putative selection event. By contrast, we used highly dynamic bar‐built estuary populations to analyse exceptional allele frequency changes within a generation, to identify the patterns of spatial and temporal repeatability in genomic outlier responses. Consistent with our prediction, repeated outlier responses occurred significantly more than expected by chance across both space and time. These findings suggest that combining spatial and temporal sampling can reveal components of putative selection that would be missed from spatial comparisons alone, including responses that are spatially shared and those that are specific to particular locations.

### 4.1 How Repeatable Is Natural Selection Over Space and Time?

By analysing within‐generation F_ST_ outliers across six estuaries in two consecutive years, we found that all repeatability types (i.e., spatial, temporal, spatio‐temporal) occurred significantly more than expected by chance, except at the maximum level of spatial repeatability (Spatial 6: 1 outlier observed, *p* = 0.15). When considering raw counts, the number of shared outliers declined as the number of estuaries increased within both the spatial and spatio‐temporal categories. For example, pairs of estuaries shared more outliers than did triplets of estuaries. Once the observed counts were standardized by their expected frequencies under the null model, however, enrichment increased with broader spatial sharing, particularly for outliers repeated across both years. Thus, exact sharing among many estuaries was numerically rare but more strongly enriched relative to random‐overlap expectations. Temporally‐repeated outliers were frequently also spatially repeated, indicating that some genomic responses that are consistent across space are also consistent through time within those same locations. Importantly, however, no outliers were spatio‐temporally repeated in more than three estuaries. We interpret this result cautiously: it does not imply a hard biological limit to repeatability but rather suggests that exact SNP‐level sharing across many estuaries in both years might be difficult to detect in a system with polygenic responses, high gene flow, heterogeneous local environments, and temporal variation in selective pressures.

If repeated local selection contributes to seasonal genomic change, we might expect estuaries with similar temporal outlier profiles to share similar ecological features that are not present in estuaries without those temporal outliers. We find some descriptive support for this possibility, although the environmental analyses did not identify the specific causal drivers. First, outlier patterns differed among estuaries in ways that were broadly consistent between years. In PC space, for example, Waddell and Scott showed very similar outlier profiles in both years (Figure 4A, Figure 4B), and the relative positions of several estuaries were qualitatively similar across the independently generated ordinations. This consistency suggests that estuary identity might mediate seasonal genomic responses, although we cannot exclude contributions from underlying population structure. Second, some environmental features vary among estuaries in ways that could plausibly influence selection. For example, Waddell and Scott are both larger than the other estuaries and both contain fish predators that are typically absent from the other estuaries (Paccard et al. 2018); previous work has linked such ecological differences to antipredator trait evolution in this system (Wasserman et al. 2020). However, our environmental‐genetic vector analysis did not detect significant relationships between seasonal genomic vectors and the measured YSI variables (Table S5). Moreover, these environmental variables likely covary and were measured at a relatively coarse temporal resolution. Additional ecological data, including predator composition and abundance, food availability, water chemistry, and finer‐scale temporal measurements, will be needed to more confidently identify the selective drivers of estuary‐specific genomic responses.

Despite the notable correspondence between outliers in space and time, many outliers were repeated across years within only a single estuary. Although the observed‐to‐expected ratio of this category was not the largest, its observed count exceeded the random‐overlap expectation by 50,250 SNPs. Thus, temporally consistent, estuary‐specific genomic responses might be common. Because this form of repeatability would not be detected by standard spatial comparisons, spatial‐only studies could underestimate the prevalence of locally consistent genomic responses. Another implication is that many consistent selection pressures might be extremely local, which—in fact–corresponds well with recent papers emphasizing that much evolution is not parallel among populations despite seemingly similar environments (Bolnick et al. 2018; Heckley et al. 2022; Oke et al. 2017) and such deviations have been associated with environmental heterogeneity (Stuart et al. 2017). This non‐parallelism should not be surprising as no two ‘similar’ natural environments are ever identical, and population‐specific environmental features could influence selection just as much as those that are shared among locations.

Interestingly, some aspects of selection differed between years in ways that were consistent in space. Across all estuaries, the 5% F_ST_ cutoff values were higher, and sharing across estuaries was greater, in 2016 than in 2017. This difference between years suggests that selection was possibly stronger and more spatially consistent in 2016 as compared to 2017. Shifts in precipitation might explain this difference. Precipitation is involved in estuary breaching, as increased rainfall and water flow help to break down the sandbar (Behrens et al. 2013, 2016). As 2016 and 2017 were dry and wet years, respectively (Table S2), the estuaries were open for equal or longer durations in all estuaries and seasons in 2017 than in 2016, except for Laguna in the fall (unpublished data, Dr. Ben Wasserman). As a result, the period during which the estuaries were closed tended to be longer in 2016 than in 2017, thus extending the time for selection to act as well as potentially increasing the strength of selection. Further, diets and diet‐related trophic morphology differed between the years, with zooplankton being more abundant in 2017 as compared to 2016 (unpublished data, Dr. Simone Des Roches). It is thus possible that in 2016, reduced precipitation and food availability (along with other factors) acted as spatially shared and stronger selective pressures. Such selective pressures could have resulted in stronger overall selection across the genome. Estimates of divergence between the marine and estuary populations and finer‐scale temporal sampling across both years could help to test this interpretation.

### 4.2 How Do the Targets of Selection Vary by Repeatability Type?

To understand how the candidate outlier responses vary over space and time, we examined the genomic position, associated candidate genes, and functional enrichment across repeatability types. The repeatability types did not show clearly distinct distribution patterns across the genome, suggesting that broadly similar genomic regions might contribute to several forms of repeatability. This pattern is consistent with previous work showing spatially‐shared seasonal genomic responses across many regions of the genome in this system (Garcia‐Elfring et al. 2021). Theory predicts that local (i.e., low spatial sharing) and global (i.e., high spatial sharing) selection result in different clustering profiles: local selection might promote tighter genomic clustering of large‐effect alleles than more spatially widespread selection, although temporal heterogeneity and low levels of gene flow can reduce these differences (Yeaman 2022). The similarity of the genomic positioning between the repeatability types might therefore reflect the high variation in selective environments across years. Higher resolution temporal sampling, for example monthly sampling through the closed‐estuary period, would help to clarify whether particular genomic regions respond to temporally consistent pressures. It is also possible that the outlier‐based approach influenced these patterns. If many adaptive responses involve alleles of small effect (Rockman 2012), focusing on the top 5% of the F_ST_ distribution might exclude many loci contributing to seasonal allele‐frequency change. If small‐effect loci contribute differently across repeatability types, this could partly homogenize the apparent clustering patterns across categories.

For each repeatability type, we detected candidate genes that map to the outliers of interest. Several candidate genes were represented by multiple outliers per repeatability type, providing stronger support that they are responding to selection. Although we detected no candidate genes across all time points and estuaries, we discovered that many candidate genes are shared across repeatability types. An average of 13.53% (sd = 4.66) of candidate genes were unique to each repeatability type, meaning that most loci are potentially under selection across multiple repeatability types. Selection acts on phenotypic function, which derives from morphological traits, their underlying loci, and the distinct genetic variants within those loci, each of which can demonstrate many‐to‐one mapping with the previous level (Bolnick et al. 2018). Many‐to‐one mapping has been found to decrease repeatability (Bolnick et al. 2018; Thompson et al. 2017). As we assessed repeatability based on the overlap of outlier SNPs, and because our populations are closely related, our estimates of repeatability would likely be higher had we investigated repeatability across loci (Conte et al. 2012). Further, our definitions of repeatability were highly restrictive, eliminating some forms of repeatability. For example, SNPs that were outliers in a given estuary in year one, but in a different estuary in year two were not considered to be repeated.

Across the shared genes, we identified 11 candidates that occurred in all estuary‐specific and temporally unique repeatability types. Several of these genes have been implicated in related biological contexts, making them useful hypotheses for future study. For example, *vma21*, which is involved in ATP synthesis (reviewed in Finbow and Harrison 1997), was found to be under selection between sympatric anadromous and lagoon resident stickleback in Scotland (Dean et al. 2019). Osmoregulatory‐related genes have been selected over various spatial scales in bar‐built estuary systems (Garcia‐Elfring et al. 2021), while genes involved in maintaining osmotic homeostasis are under positive selection for estuarine Atlantic killifish (Reid et al. 2017). *erbb4b* is likely involved in salinity tolerance in stickleback populations, possibly through physiological stress responses (Kusakabe et al. 2017). *grin2ab* and *PLCB1* are associated with signalling pathways, with the former being upregulated in socially housed stickleback (Greenwood and Peichel 2015), and the latter under selection in migratory anadromous Hilsa shad (Asaduzzaman et al. 2020). These candidate genes are therefore broadly consistent with existing literature, but they should be interpreted as hypothesis‐generating rather than as confirmed targets of selection. Several Manhattan plots showed a concentration of outliers on chromosome 4, which has repeatedly been implicated in freshwater adaptation in stickleback (Hohenlohe et al. 2010; Bassham et al. 2018). However, because our clustering analyses provided mixed support and as we did not test whether these outliers overlap with the same haplotypes identified in previous studies, we treat this correspondence as a qualitative observation and hypothesis for future work. Future experimental or individual‐level genomic work will be needed to determine the causal variants, mechanism, selective pressure, phenotypic effect, and consistency of responses across the populations, especially as each mapped SNP does not necessarily have a phenotypic effect.

Across our candidate loci, we performed functional enrichment analyses for each repeatability type and detected unique and repeated functions. For example, organic acid metabolic activity and appendage‐related development were unique to Old Dairy and Laguna, respectively (Table S16). This indicates that particular functions might be under local selection; however, additional experimental analyses are needed to identify the source, strength, and consistency of potential local pressures. Across repeatability types, enrichment of function related to nervous‐system development, cell migration/organization, and anatomical/tissue development appeared to be highly repeated. This indicates that these functions are likely under repeated selection over space and time, despite not necessarily involving identical SNPs and/or loci.

## 5 Conclusion

In this study, we leveraged the dramatic environmental variation created by seasonal breaching of estuary sandbars to test the parallelism of natural selection across space and time. Extensive gene flow between estuaries and the ocean likely helps to maintain phenotypic and genotypic variation, contrasting with a more typical focus on well‐adapted and relatively isolated populations. In such stable systems, contemporary measures of selection might reveal weaker or less widespread responses than historical patterns of adaptive divergence. Experimental studies of selection often address this problem by creating well‐mixed populations that combine distinct genotypes and then introduce them to similar environments (e.g., Barrett et al. 2019; Berardi et al. 2025). The Santa Cruz estuary system provides a natural analogue, allowing us to examine repeated seasonal allele frequency change without experimental manipulation. We found that, even at the level of SNPs, seasonal genomic outlier responses were spatially and temporally repeated more often than expected by chance. Although repeatability occurred most frequently across limited numbers of populations, enrichment relative to null expectations was greatest when greater numbers of locations and/or multiple time points were considered. Temporally repeated responses were also prominent, highlighting the value of repeated temporal sampling for detecting local components of putative selection that would be missed by spatial comparisons alone. We also found that repeatability was greater at the gene level than at the SNP level, indicating that our SNP‐based estimates are likely highly conservative. Future work should assess spatial and temporal repeatability at higher levels of biological organization, including physiology and morphology, particularly in wild populations. Our findings suggest that the genomic variation across populations reflects a complex mix of idiosyncratic, local, spatially broad, and temporally consistent selection pressures, providing insight into the parallel and non‐parallel features of adaptive evolution.

## Author Contributions

Study design was conceptualized by Eric P. Palkovacs, Andrew P. Hendry and Rowan D.H. Barrett. Tissue and ecological data collection were performed by Ben A. Wasserman. Molecular extractions and quantification were performed by Alan Garcia‐Elfring and Mary Kathleen (MK) Hickox. Bioinformatics and statistical analysis were completed by Mary Kathleen (MK) Hickox with guidance from Alan Garcia‐Elfring, Ben A. Wasserman and Rowan D.H. Barrett. Alan Garcia‐Elfring wrote and performed the clustering analyses. The manuscript was written by Mary Kathleen (MK) Hickox with feedback from all authors.

## Funding

Mary Kathleen Hickox was supported by the Natural Sciences and Engineering Research Council of Canada (NSERC, 54310‐32019) Graduate Scholarship, Fonds de recherche du Québec (FRQNT, 298356) Doctoral Training Scholarship, and Mitacs Globalink Research Award (IT25595). Andrew P. Hendry, and Rowan D. H. Barrett were supported by NSERC Discovery Grants (543103‐2019, RGPIN‐2018‐04761, RGPIN‐2019‐04549) and Canada Research Chairs (246984). Eric P. Palkovacs was supported by the NOAA Cooperative Institute for Marine, Earth and Atmospheric Systems (CIMEAS).

## Ethics Statement

Sampling was performed according to the California Scientific Collector's Permit SC‐12752. All fish collection and handling procedures were in accordance with the University of California, Santa Cruz IACUC protocols Palke‐1306 and Palke‐1310. We occasionally used Co‐pilot (GPT‐4.1) to assist with code optimization; all code and outputs were checked by the authors.

## Conflicts of Interest

The authors declare no conflicts of interest.

## Supporting information


**Figure S1:** Manhattan plots showing F_ST_ values across the genome in each estuary/year. The 5% cutoff value is represented by the dashed line, so that genomic sites above the line are outliers. The genomic sites are coloured according to repeatability type for the outliers that are spatially repeated.
**Figure S2:** Manhattan plots of the sliding window F_ST_ values in each estuary/year, plotted at the window midpoint. F_ST_ was calculated in 50,000 base pair sliding windows with 10,000 base pair overlap. The 5% cutoff value is represented by the dashed line, so that genomic sites above the line are outliers. The genomic sites are coloured according to outlier status.

**Table S1:** A hypothetical example demonstrating repeatability types.
**Table S2:** Environmental data relating to the estuaries (precipitation).
**Table S3:** Pool size (i.e., the number of individual stickleback per pool).
**Table S4:** Repeatability type observed, 1% and 10% Cutoff.
**Table S5:** Results from the vector analysis.
**Table S6:** Ripley's K.
**Table S7:–15** Genic matches for each of the focal datasets.
**Table S16:** Full GO lists.

**Data S1:** Methods S1. Vector analysis.
**Methods S2**. Clustering tests.
**Methods S3**. Probability by repeatability type.

## Data Availability

The raw 2016 and 2017 data are available at the National Center for Biotechnology Information (NCBI) Sequence Read Archive (PRJNA704280) and (PRJNA1347114), respectively. Scripts are available on GitHub (https://github.com/mk‐hickox/Spatial_Temporal_Repeatability).
