# Supplementary material for: The Spatial and Temporal Repeatability of Genomic Responses to Natural Selection as Demonstrated in Stickleback Populations Experiencing Highly Dynamic Environments
Source: Mol Ecol. 2026 Sep 4;35(17):e70528. doi: 10.1111/mec.70528 (PMC13545515; doi:10.1111/mec.70528)
Supplement: Supplementary file 1 [file MEC-35-e70528-s001.docx]

**Supplementary Methods 1, Vector Analysis:**

To further examine how repeated selective responses relate to environmental variation and gene flow, we ran a vector analysis, following the methods of Stuart et al. 2017. We ran the analysis within each year (i.e., 2016 and 2017 separately) using datasets of the neutral SNPs, outlier SNPs, and environmental YSI data averaged per site (dissolved oxygen (mg/L), salinity (ppt), and temperature (°C). Neutral SNPs are genetic sites that are not outliers in any year or estuary, whereas outlier SNPs are 5% outliers in at least one estuary of the given year of analysis. We standardized the data by generating z-scores of the means for environmental data, and by running PCAs on the allele frequency data (as described in Methods 2.2). For all genetic analyses, we included the first four principal component axes, as they represented a cumulative variance explained of >50% in each dataset.

We generated the vectors between the seasons of each population (estuary-year), representing our period of selection. The length of the seasonal vector of each population thus represents the magnitude of seasonal-divergence (L) over multivariate space. The direction of the seasonal vector represents the relative contribution of different variables over multivariate space. As such, the angle between the seasonal vectors of different populations, or theta (Θ), represents the parallelism of the responses (e.g., Θ=0= parallel or repeated seasonal response). We additionally calculated the absolute difference in the magnitude of seasonal-divergence (ΔL) between each population. To understand whether seasonal selective responses are more similar for estuaries with similar seasonal environmental characteristics, we ran Mantel tests with 9999 permutations for ΔL and Θ, using the outlier SNP and environmental datasets. To understand the role of gene flow across the system, we ran the same analysis using the neutral SNP and environmental datasets. We then ran linear models to understand whether the magnitude of seasonal-divergence of selection (as measured via the outlier SNPs) related to that of the environment (as measured via the YSI data) or gene flow (as measured via the neutral SNPs). For additional details on the analysis, please consult Stuart et al. (2017).

**Supplementary Methods 2, Clustering Tests:**

We tested for spatial clustering of outlier SNPs using Ripley’s K, as implemented in a custom R script (v6.2). Ripley’s K-function characterized spatial point patterns across chromosome-specific distance scales using the one-dimensional translation edge correction of Ohser (Ohser, 1983; Ripley, 1976). K-functions were evaluated at 20 distance increments per chromosome, with minimum distances determined by mean SNP spacing and maximum distances capped at either 500 kb or one-quarter of chromosome length. K-values were transformed using L(r) − r, where positive deviations indicate clustering and negative deviations indicate regular spacing. Chromosomes containing more than 5,000 SNPs were randomly sub-sampled to 5,000 SNPs prior to K-function computation to improve computational efficiency.

The analyses employed a density-preserving block-permutation null model (Baddeley et al., 2016). Under the block-permutation approach, chromosomes were divided into contiguous 100-kb blocks and SNP positions were randomized uniformly within each block while preserving block-level SNP counts. This procedure maintains broad-scale SNP density variation while testing for clustering relative to local genomic context (Nordborg & Tavaré, 2002). Statistical significance was assessed at α = 0.01 using permutation tests (499 replicates for Ripley’s K), with 99.5% permutation envelopes used for Ripley analyses.

**Supplementary Methods 3, Probability by Repeatability Type:**

**Spatial** $= \binom{6}{k}\times{2\times0.05^{k}\times(1-0.05){}^{6-k}}\times(1-0.05)^{6}$

A spatially repeated SNP is a 5% outlier in two or more estuaries in the same year, but not a 5% outlier in any estuary in the other year.

| $\binom{6}{k}$ | Of the six estuaries, the number of possible combinations of estuaries that share an outlier, with sharing being possible between two to six estuaries (k=2:6) in a given year |
| --- | --- |
| $2\times$ | We double the value since we are interested in the probability of spatial outliers over both years (i.e., 2016 and 2017) |
| $0.05^{k}$ | The probability that the SNP is an outlier for “k” estuaries in a given year. As we studied six estuaries, the outlier could be spatially shared between two to six estuaries (k=2:6) |
| $(1-0.05){}^{6-k}$ | The probability that the SNP is not an outlier for the remaining estuaries of that year (i.e., if we are interested in spatial 2, we are calculating the probability that it is not an outlier in the other four estuaries) |
| $(1-0.05)^{6}$ | The SNP is not an outlier in any of the estuaries in the alternate year (i.e., if the SNP is an outlier in 2016, it is not an outlier in any of the six estuaries in 2017) |

**Temporal** $= 6\times0.05^{2} \times(1-0.05^{2})^{5}$

A temporally repeated SNP is a 5% outlier in both 2016 and 2017 in the same estuary, and only for that estuary.

| $6\times$ | We multiply the value by six since we are interested in the probability of temporal outliers over all six estuaries |
| --- | --- |
| $0.05^{2}$ | The probability that the SNP is an outlier in both years for the estuary |
| $(1-0.05^{2})^{5}$ | The probability that the SNP is not an outlier in both years of the other five estuaries |

**Spatio-temporal** =$\binom{6}{k}{\times(0.05^{2})^{k}\times(1-0.05^{2}){}^{6-k}}$

Spatio-temporally repeated SNPs are 5% outliers in both years in the same estuary for multiple estuaries.

| $\binom{6}{k}$ | Of the six estuaries, the number of possible combinations of estuaries that share an outlier, with sharing being possible between two to six estuaries (k=2:6) |
| --- | --- |
| $(0.05^{2})^{k}$ | The probability that the same SNP is an outlier in both years for a given estuary, and that this occurs for multiple estuaries (i.e., if we are interested in spatio-temporal 2, the chance that the same SNP is an outlier in both years for a given estuary, for two estuaries) |
| $(1-0.05^{2}){}^{6-k}$ | The probability that the SNP is not an outlier in both years of a given estuary, for the remaining estuaries (i.e., if we are interested in spatio-temporal 2, the chance that the SNP is not an outlier in both years for each of the remaining four estuaries) |

Assumptions:

- All genomic sites were independent
- Each genomic site had a 5% chance of being an outlier

REFERENCES

Baddeley, A., Rubak, E., & Turner, R. (2016). Spatial Point Patterns: Methodology and applications with R (Vol. 1). CRC Press, Taylor & Francis Group.

Nordborg, M., & Tavaré, S. (2002). Linkage disequilibrium: what history has to tell us. Trends in Genetics, 18(2), 83–90. 10.1016/s0168-9525(02)02557-x

Ohser, J. (1983). On estimators for the reduced second moment measure of point processes. Series Statistics, 14(1), 63–71. https://doi.org/10.1080/02331888308801687

Ripley, B. D. (1976). The second-order analysis of stationary point processes. Journal of Applied Probability, 13(2), 255–266. doi:10.2307/3212829

Stuart, Y. E., Veen, T., Weber, J. N., Hanson, D., Ravinet, M., Lohman, B. K., Thompson, C. J., Tasneem, T., Doggett, A., Izen, R., Ahmed, N., Barrett, R. D. H., Hendry, A. P., Peichel, C. L., & Bolnick, D. I. (2017). Contrasting effects of environment and genetics generate a continuum of parallel evolution. Nature Ecology & Evolution, 1(6), 0158. https://doi.org/10.1038/s41559-017-0158
