# Supplementary material for: The Spatial and Temporal Repeatability of Genomic Responses to Natural Selection as Demonstrated in Stickleback Populations Experiencing Highly Dynamic Environments
Source: Mol Ecol. 2026 Sep 4;35(17):e70528. doi: 10.1111/mec.70528 (PMC13545515; doi:10.1111/mec.70528)
Supplement: Supplementary file 2 [file MEC-35-e70528-s003.docx]

*Supplementary Figure 1*. Manhattan plots showing F_ST_ values across the genome in each estuary/year. The 5% cutoff value is represented by the dashed line, so that genomic sites above the line are outliers. The genomic sites are coloured according to repeatability type for the outliers that are spatially repeated.

*Supplementary Figure 2*. Manhattan plots of the sliding window F_ST_ values in each estuary/year, plotted at the window midpoint. F_ST_ was calculated in 50,000 base pair sliding windows with 10,000 base pair overlap. The 5% cutoff value is represented by the dashed line, so that genomic sites above the line are outliers. The genomic sites are coloured according to outlier status.
